# Generation of decellularized human brain tissue for investigating cell-matrix interactions: a proof-of-concept study

**DOI:** 10.3389/fbioe.2025.1578467

**Published:** 2025-06-05

**Authors:** Roemel Jeusep Bueno, Camila Fernández-Zapata, Maren Salla, Juliana Campo Garcia, Aylin Alacam, Oliver Klein, Chotima Böttcher, Helena Radbruch, Friedemann Paul, Sarah C. Starossom, Rafaela V. Silva, Carmen Infante-Duarte

**Affiliations:** ^1^ Experimental and Clinical Research Center, A Cooperation Between Max Delbrück Center and Charité Universitätsmedizin Berlin, Berlin, Germany; ^2^ Faculty of Life Sciences, Humboldt-Universität zu Berlin, Berlin, Germany; ^3^ Berlin School for Regenerative Therapies (BSRT), Charité – Universitätsmedizin Berlin, Berlin, Germany; ^4^ Max-Delbrück-Center for Molecular Medicine (MDC), Berlin Institute for Medical Systems Biology (BIMSB), Berlin, Germany; ^5^ Berlin Institute of Health at Charité Universitätsmedizin Berlin, Berlin, Germany; ^6^ Department of Biology, Chemistry, Pharmacy, Freie Universität Berlin, Berlin, Germany; ^7^ Berlin Institute of Health (BIH), Center for Regenerative Therapies, Berlin, Germany; ^8^ Department of Neuropathology, Charité-Universitätsmedizin, Berlin, Germany

**Keywords:** brain extracellular matrix, matrisome, neuronal stem cells, monocytes, brain proteomics, imaging mass cytometry

## Abstract

The brain extracellular matrix (ECM) regulates myelin repair and regeneration following a demyelinating event by interacting with neuronal progenitors and immune cells. Therefore, generation and characterization of decellularized human brain tissue (DHBT) in regions with distinct neuroregenerative capacities are essential to determine factors modulating the cellular regenerative behavior. We have established an effective decellularization protocol for the human neural stem cell (NSC)-rich subventricular zone (SVZ) as well as, frontal cortex (FC) and white matter (WM), and defined region-specific matrisomes with comparative proteomics. Subsequently, as proof-of-concept, survival and differentiation of NSCs and monocytes within the DHBT were investigated. The proteomic analysis of the DHBT confirmed the retention of matrisome proteins such as COL4A1, FBB, NCAN, ANXA2. Unique to the SVZ were LGI3 and C1QB, while annexins, S100A and TGM2 were found in FC; S100B was exclusive to the WM. NSCs cultured within WM and FC acquired an astrocytic phenotype, but both astrocytic and oligodendrocytic phenotypes were promoted by the SVZ DHBT. Moreover, imaging mass cytometry analysis indicated an anti-inflammatory phenotype differentiation of monocytes seeded on SVZ and WM. Thus, the established model is suitable for investigation of ECM properties and assessment of cell-matrix interactions.

## 1 Introduction

The brain extracellular matrix (ECM) constitutes approximately 20% of the total brain volume (Tarricone et al., 2022). It is composed of a highly organized and complex meshwork of glycosaminoglycans and proteins, principally collagens, as well as glycoproteins, proteoglycans, and other associated and secreted proteins. The brain ECM is essential for homeostatic and regenerative processes in the central nervous system (CNS) (Busch and Silver, 2007; Bonneh-Barkay and Wiley, 2009; Mendibil et al., 2020), influencing cell migration, polarity and differentiation as well as axonal and neurite growth, and synaptogenesis (Lau et al., 2013; Melrose et al., 2021).

ECM scaffolds are biomaterials that can be generated by tissue decellularization and can be used to investigate ECM-cell interactions and to characterize matrisome proteins, a group of core ECM and ECM-associated proteins (Granato et al., 2020; Yaldiz et al., 2022). However, most of the studies on decellularized brain sections employed animal material and the knowledge on human scaffolds is very limited (DeQuach et al., 2011; De Waele et al., 2015; Segel et al., 2019; Granato et al., 2020; Terek, 2023). Moreover, translating animal research on brain scaffolds to the human situation can be difficult, since the ECM composition appears to differ between human and animals with smooth surface brains, such as the mouse (Fietz et al., 2012). At the proteome level, interspecies variation accounts for about 40% of discovered matrisome proteins (Pokhilko et al., 2021). Thus, by establishing an accurate protocol to decellularize human brain tissue, we aimed to create a more relevant and human-specific model to closely mimic the native microenvironment of the CNS and decipher the intricate interplay between ECM alterations and neuroinflammatory responses in demyelinating diseases. Moreover, generation of decellularized human brain tissue (DHBT) holds a great potential for investigating the influence of ECM on cellular differentiation during CNS regeneration and repair processes.

It is well established that CNS injury or demyelination can induce alterations in the composition of the ECM (Dityatev et al., 2021; Soles et al., 2023), generating an environment that can be permissive or suppressive to CNS regeneration and repair (de Jong et al., 2020; Silva et al., 2023). In this context, both peripheral immune cells such as monocytes (Starossom et al., 2019; Forbes and Miron, 2022; Campo Garcia et al., 2024) and brain resident neural stem cells (NSCs) (Sanai et al., 2005; De Gioia et al., 2020; Campo Garcia et al., 2024), have been considered as main players in regenerative processes following demyelination, and both cell types are believed to be modulated by local ECM stimuli.

In the mammalian brain, the subventricular zone (SVZ) is a region that contains a stem cell niche constituted by progenitor cells such as NSCs and oligodendrocyte precursor cells (OPCs), which contribute to myelin repair in demyelinating diseases (Nait-Oumesmar et al., 2008; Butt et al., 2022; Donega et al., 2022). Thus, it is crucial to determine the peculiarities of the ECM in this region with regenerative activity in comparison with the ECM of areas in which active demyelination may occur, such as frontal cortex and white matter parenchyma (Amin and Borrell, 2020; Lie et al., 2022).

In this proof-of-concept study, we established a chemical-enzymatic decellularization protocol to generate DHBTs of human stem cell (SVZ) and non-stem cell niche (frontal cortex, white matter) areas. The region-specific DHBTs were then characterized using a proteomic approach. We hypothesize that ECM from the SVZ will possess elements that modulate cell differentiation toward a regenerative phenotype. Thus, we tested and compared exemplarily the suitability of the DHBTs to modulate cellular differentiation in monocytes and neuronal progenitor cells *ex vivo*.

## 2 Materials and methods

### 2.1 Human brain tissue acquisition and preparation

Autopsy was performed at the Department of Neuropathology at Charité Universitaetsmedizin Berlin. The collection of human brain samples and subsequent use for this study was approved by the local ethics committee (EA1/368/20 and EA1/144/13) and following a structured informed consent process. The use of human samples in this study conforms to all relevant national regulations, institutional policies, and is in accordance with the tenets of the Helsinki Declaration. The human brain specimens included in this study were sections from frontal cortex, SVZ, and normal appearing white matter. The sample donor was an 84-year-old female with progressive supranuclear palsy (PSP) confirmed by neuropathological workup. The neuropathological examination during the autopsy was performed at the Department of Neuropathology at Charité Universitätsmedizin Berlin. At least one certified neuropathologist identified the anatomical regions and collected all samples included in this study from the brain without previous fixation. First, the gyri were removed and cryopreserved on dry ice, then the unfixed brain was cut into coronal, 1 cm thick sections on a cooled table. The subventricular zone of the frontal horn of the lateral ventricles was identified and sampled on the level corresponding to Plate 12 with caudate nucleus and corpus callosum. The middle frontal gyrus was identified and sampled on the level corresponding to Plate 3 of the Human Brain Atlas (Mai and Voss, 2009). The normal appearing white matter was identified and sampled in the frontal lobe with at least 1 cm distance to any lesion. All samples were cryopreserved and slowly frozen on dry ice and stored at −80°C prior to cryosectioning. The native brain samples were cut by cryosectioning in 200 µm-thick slices onto 8-well culture chamber slide (Cat.No: 80841, Ibidi, Germany) and stored at −80°C until further processing.

### 2.2 Decellularization of post-mortem human brain tissue slices

To produce DHBTs, 3 different preparation protocols were devised. All steps were performed in an 8-well culture chamber slide under sterile conditions. All decellularization strategies started by washing with cold PBS (1x, without Mg^2+^/Ca^2+^) (Gibco, Thermo Fischer Scientific, United States) with 1% antibiotic/antimycotic (A/A) (Gibco, Thermo Fischer Scientific, United States). The slices were then incubated with deionized water for 30 min. The different decellularization protocols were done with different incubation periods of 0.5% sodium deoxycholate (SDC) (Sigma-Aldrich, United States) and 1 mg/mL DNase I (Merck, Germany) dissolved in PBS (1x, with Mg^2+^/Ca^2+^) with 1% A/A. There was an additional washing step with PBS (1x, with Mg^2+^/Ca^2+^) with 1% A/A in-between the incubation with SDC and DNase I. The first protocol (P1) consisted of 20-min incubation with 0.5% SDC and 1-h incubation with DNase I. The second protocol (P2) was 30-min incubation with 0.5% SDC and 30-min incubation with DNase I. The third protocol (P3) includes a 30-min incubation with 0.5% SDC and 1-h incubation with DNase I. All decellularization protocols were concluded by a 15 min incubation with 0.15% SDC followed by three washes with PBS (1x, without Mg^2+^/Ca^2+^) containing 1% A/A. For the optimization of the three decellularization protocols (P1, P2, and P3), normal appearing white matter brain tissue was used. For all succeeding decellularization of the three brain regions (frontal cortex, subventricular zone, and normal appearing white matter), the P1 protocol which consists of 20-min incubation of 0.5% SDC and 1-h incubation of DNase I was applied.

### 2.3 DNA extraction and quantification

DNA was isolated and quantified from brain samples decellularized using the three different protocols mentioned above (P1, P2, P3) and non-decellularized brain samples of normal appearing white matter. DNA was isolated using the DNA Mini Kit (QIAGEN, Germany) in accordance with manufacturer’s specifications. The DNA concentration in the extracts was measured using the Thermo Scientific NanoDrop 2000/2000c Spectrometer (Thermo Fisher Scientific, United States).

### 2.4 Proteomic sample preparation

The DHBTs were prepared for proteomics through lyophilization. After decellularization, two slices from each of the three brain regions were ground in a frozen mortar and pestle placed on dry ice. Once the samples were in frozen powder form, they were collected with sterile deionized water onto pre-weighed 1.5 mL low-binding Eppendorf tubes. The tubes, which were previously punctured with holes on top, were then placed on a Steriflip-GP tube (Merck, Germany). The samples were then lyophilized using a freeze dryer overnight at 0.01 mBar and −50°C. Once dried, the samples were weighed again prior to proteomic measurement. Non-decellularized control from native white matter tissue was processed for proteomics as a basis of comparison. All samples weighed at least 5 mg prior to proteomic measurement.

### 2.5 Nano-liquid chromatography-tandem mass spectrometry (NanoESIQTOF-LC-MS/MS) and proteomic data analysis

Approximately 0.05 mg of sample was injected into a NanoHPLC (Dionex UltiMate 3000, Thermo Fisher Scientific, United States) coupled to an ESI-QTOF ultra high-resolution mass spectrometer (Impact II, Bruker Daltonic GmbH, Germany) as described previously (Daneshgar et al., 2020).

In brief, the samples were loaded onto the Acclaim PepMap 100 Nano-Trap column (Thermo Scientific P/N 164564, Thermo Fisher Scientific, United States) and eluted after calibration with an increasing gradient. Peptides were separated on a 75 μm by 50 cm Acclaim PepMap RSLC column packed with silica (Thermo Scientific 164939, Thermo Fisher Scientific, United States) and subsequently detected by mass spectrometry. A full-mass scan (150–2,200 m/z) was performed at a resolving power of 50,000. The auto MS/MS InsantExpertise nanobooster was used to select peaks for fragmentation by Collision-induced dissociation. The derived peak lists were analyzed against the human Swiss-Prot database using PEAKS studio proteomics software version 10.6 (Bioinformatics Solutions, Waterloo, Canada). The analysis was conducted with the default settings of PEAKS Studio 10.6, without merging the scans. The correct precursor was identified using mass only. Peptide identifications were carried out within PEAKS using its proprietary search engine, PEAKS DB, in combination with PEAKS *de novo* sequencing. The PEAKS PTM search tool was utilized to identify unspecified peptides homologous to those in the protein database. The default maximum number of variable post-translational modifications per peptide was set to three. The retention time shift tolerance was 1 min. All search tools are integrated within the PEAKS Studio software. The false discovery rate (FDR) was calculated using target decoy fusion and set to 0.01. The mass spectrometry proteomic data have been deposited to the ProteomeXchange Consortium via the PRIDE partner repository with the dataset identifier PXD062743.

To identify the detected matrisome proteins, the dataset obtained from the proteomic measurement was compared to the data platform MatrisomeDB (Naba et al., 2016; Shao et al., 2020). The matrisome proteins were categorized into 5 different groups, namely: collagens, ECM glycoproteins, proteoglycans, ECM regulators, ECM-affiliated proteins, and secreted factors.

### 2.6 Immunohistochemical staining and imaging

The DHBTs were fixed with 4% paraformaldehyde (PFA) and then washed with PBS before proceeding to cryosectioning. The fixed samples and submerged in OCT and sectioned into 10 µm slices for staining. The slices were blocked for 1 hour at room temperature with 5% BSA and 5% Horse serum in PBS. Primary antibodies including Chondroitin Sulfate (CS) (1:200, C8035-100UL, Sigma-Aldrich, United States), Collagen IV (Col IV) (1:500, ab236640, Abcam, UK), Fibronectin (1:150, ab23750, Abcam,UK), Heparan Sulfate (HS) (1:1000, 370255-1, Amsbio, UK) and Laminin alpha-1 (Lama1) (1:100, ab11575, Abcam, UK) were incubated in blocking solution at 4°C overnight. Secondary antibodies including goat anti-mouse IgM conjugated with Alexa Fluor 488 (1:500, A-21042, Thermo Fischer Scientific, United States) and donkey anti-rabbit IgG conjugated with Alexa Fluor 555 (1:500, A-31572, Thermo Fischer Scientific, United States) were incubated in diluted blocking solution (8% horse serum, 3% BSA, and 0.3% triton) for 1 hour at room temperature. All samples were counter-stained with 4′,6-diamidino-2-phenylindole (DAPI, 1:10000 dilution, Sigma-Aldrich, United States). A negative control was also treated parallel to every staining cycle and treated identically with the exception of the addition of a primary antibody. The slices were washed with PBS after staining and mounted on a glass slide with Immu-Mount (Thermo Fisher Scientific, United States) for imaging. The samples were imaged using a Zeiss LSM 700 with the ×40 objective, in confocal mode.

### 2.7 Seeding of neural stem cells on decellularized human brain tissue

After preparation of the sterile DHBTs on 8-well culture chamber slides, they were incubated overnight in NSC differentiation medium composed of KnockOut DMEM/F-12, 2% StemPro Neural Supplement, 2 mM Glutamax, 1% Antibiotic/Antimycotic (Gibco, Thermo Fischer Scientific, United States). The following day, the medium was removed. NSCs derived from the human pluripotent stem cell line H9 (purchased from Thermo Fischer Scientific, United States), and at stem cell passage 25, were seeded onto the DHBTs as a condensed droplet at a density of 1 × 10^5^ cells and incubated overnight with StemPro Serum Free Proliferation Medium (KnockOut DMEM/F-12, 2% StemPro Supplement, 10 ng/mL EGF, 10 ng/mL FGF2, 2 mM GlutaMAX, and 1% A/A). A droplet of same density was also seeded onto glass coated with Laminin (Thermo Fisher Scientific, United States) as a control. The next day, the medium was removed and replaced with NSC differentiation medium. After 8 days of differentiation, the medium was removed and the recellularized ECM were either fixed and stained for immunohistochemistry or lysed in preparation for RNA processing and quantification.

### 2.8 RNA quantification and real-time quantitative PCR (qPCR)

RNA was isolated and quantified from native non-decellularized brain samples and from decellularized samples with and without seeded cells. RNA was isolated using the RNeasy Micro Kit (QIAGEN, Germany) in accordance with manufacturer’s specifications. The RNA concentration in the extracts was measured using the Thermo Fisher Scientific Qubit with RNA High Sensitivity Kit (Thermo Fisher Scientific, United States). The list of primers used for the qPCR is provided in Supplementary Table S1. A two-step qPCR protocol was performed using the High-Capacity cDNA Reverse Transcription Kit (Thermo Fisher Scientific, United States) and the TaqMan Fast Advanced Mastermix (Applied Biosystems, United States) with 5 μL reaction system according to the manufacturer’s protocol by QuantStudio Real-Time PCR Systems (Thermo Fisher Scientific, United States). Each reaction was performed in triplicates and normalized to the B2M gene expression. The CT value of each well was determined using the QuantStudio Real-Time PCR System software. The relative quantification was determined by arbitrary units (2^−ΔCT^).

### 2.9 Isolation of peripheral blood mononuclear cells (PBMCs) from leukocyte enriched buffy coats and isolation of CD14^+^ monocytes from PBMCs

Leucocyte-enriched buffy coat from an anonymous healthy donor (female, 40) was obtained from the German Red Cross (DRK) through a structured informed consent process of blood donation. For the isolation of PBMCs, buffy coat content was diluted with an equal volume of PBS and centrifuged with a density gradient medium (density of 1,007 g/mL) for 20 min at 760 x g at room temperature. The PBMC ring was carefully removed, washed in wash medium (5% FCS in RPMI 1640 containing 1% Hepes), resuspended in freezing medium (20% DMSO, 20% FCS, RPMI 1640 containing 1% Hepes) at a density of 5 × 10^6^ cells/mL and stored in liquid nitrogen until further use. Upon experimental use, PBMCs were thawed, resuspended in thawing medium (10% FCS, RPMI1640) and counted by trypan blue exclusion. Both dead and live cells were counted to measure viability, on average above 95% after thawing. Monocytes were then isolated from thawed PBMCs via positive immunomagnetic selection using human CD14 MicroBeads (Miltenyi Biotec, Germany) according to manufacturer’s instructions with the exception that only 50% of the recommended bead concentration was used. Following cell sorting, cells from both positive and negative fractions were counted via trypan blue exclusion and proceeded to phenotyping analysis. Cells from pre-MACS PBMCs and post-MACS fractions (positive and negative) were blocked in FC blocking solution (Miltenyi Biotec, Germany) for 15 min at 4°C. After washing with PBS, the cells were stained with a LIVE/DEAD Fixable Near-IR viability staining (1:1000, L34975, Thermo Scientific, United States) according to manufacturer instructions, followed by staining with the following antibodies: CD14 (FITC, 1:50, 555397, BD Biosciences, United States), Lineage cocktail (CD3, CD19, CD20, CD56) (APC, 1:10, 363601, BioLegend, United States), CD40 (AF700, 1:100, 561208, BD Biosciences, United States), CD69 (PE-Cy7, 1:100, 557745, BD Biosciences, United States), CD80 (BV786, 1:50, 564159, BD Biosciences, United States), CD86 (BV605, 1:100, 562999, BD Biosciences, United States), HLA-DR (PE, 1:20, 555812, BD Biosciences, United States). Cells were then incubated for 15 min at room temperature. After washing, the cells were measured on a BD Fortessa Flow Cytometer (BD Biosciences, United States).

### 2.10 Seeding isolated peripheral monocytes on decellularized human brain tissue

The DHBTs were prepared on 8-well culture chamber slides under sterile conditions and incubated overnight in serum-free OPC Spontaneous Differentiation Media Kit (Sigma-Aldrich SCM106), as previously described (Campo Garcia et al., 2024). The following day, the medium was removed, and freshly isolated peripheral monocytes were seeded onto the DHBTs as a condensed droplet at a density of 1 × 10^6^ cells and incubated with serum-free OPC Spontaneous Differentiation Media. After 48 h of incubation, the medium removed and the recellularized ECM were fixed with 4% PFA and were prepared for cryosectioning in 10 µm-thick slices onto ionized glass slides and stored at 4°C until further processing for immunohistochemistry and imaging mass cytometry.

### 2.11 Imaging mass cytometry and data analysis

Imaging mass cytometry was performed with a validated antibody panel of 15 markers. Antibodies that were not available as metal-labeled were conjugated in house using the MaxPar antibody conjugation kit according to the company’s recommendations (Fluidigm, Standard BioTools, United States). Recellularized ECM from frontal cortex, subventricular zone, and white matter were cut into 10 μm-thick sections, mounted on glass slides and stored at −80°C. For staining, the sections were first brought to room temperature (RT) and blocked for unspecific binding with 3% purified BSA in 0.1% Triton-X PBS for 30 min at RT. Sections were then incubated overnight at 4°C with mouse anti-chondroitin sulfate antibody (1:200) followed by a secondary antibody staining with 172 Yb goat anti-mouse antibody (1:100) for 3h at RT. After washing, all slides were incubated overnight at 4°C with an antibody mix, including the rest of the metal-conjugated primary antibodies (Supplementary Table S2). Nuclei were detected using an Ir-Intercalator (1:1000). Samples were then dried and stored at RT until measurement. IMC acquisition was performed on a CyTOF2/upgraded to Helios specifications coupled to a Hyperion Tissue Imager (Fluidigm, Standard BioTools, United States), as described previously (Bottcher et al., 2020).

Raw data was stored as.mcd files as well as.txt files. Original files were opened with MCD viewer, and single 16-bit images were extracted as.tiff files. For visualization only, images were transferred to ImageJ and the different channels were merged. Single cell analysis of IMC data was performed following an adapted workflow (Windhager et al., 2023). In brief, for single-cell segmentation, images were processed using Ilastik software (Ilastik Team, Germany) (Berg et al., 2019), which uses interactive machine learning to create segmentation masks distinguishing single-cells from background. The program was trained to identify DNA iridium-intercalator as nuclei and membrane markers including CD14, CD45, CD68 or CD74 as cytoplasm, while the rest was labelled as background. As a result, a binary mask delimiting each single cell was obtained and transferred on to CellProfiler (Cimini Lan, US) (Stirling et al., 2021). We then applied a set of modules including filters for maximum and minimum cell size, negative selection for cells on the border of the image or exclusion of cytoplasm signal with no nuclei, finally generating 16-bit.tiff single-cell masks with only full cells for each image. The single-cell masks and corresponding multi-channel images were then loaded into R (R Foundation for Statistical Computing, Austria) for further analysis. As a first step we used IMCRtools package (Windhager et al., 2023) to extract object specific features, including channel intensity for each of the markers and their location, which were then used to build spatial single-cell objects.

As part of the pre-processing, compensation for signal spillover was done using the CATALYST package (Chevrier et al., 2018). Batch effect correction was used to integrate measurements done on different slides using the fast mutual nearest neighbors (fastMNN) algorithm (Haghverdi et al., 2018). The multi-dimensional single-cell data was visualized in the 2-dimensional space using Uniform Manifold Approximation and Projection (UMAP) for dimensionality reduction. Identification of the different cell populations in the samples was performed using the flowSOM algorithm for clustering (Van Gassen et al., 2015). In this case the clustering was done using macrophage markers as input (Supplementary Table S2), the final number of clusters was determined using the “elbow” criterion based on the area under the CDF curve. The resulting clusters were used for statistical analysis to detect changes in frequency and marker expression between groups.

### 2.12 Statistical analyses

Microsoft Excel Office 365 (Version 15.24, Microsoft, Redmond, WA 98052, United States) and GraphPad Prism Version 8.1.1 (GraphPad Software, United States) were used for statistical analysis. All statistical analysis was performed using the technical replicates with n stated in the results section. Quantitative results are reported as means with standard deviation (SD). Group comparisons were performed using Turkey multiple comparison analysis. P-values below 0.05 were considered statistically significant.

## 3 Results

### 3.1 Establishment of the decellularization protocols on post-mortem human brain tissue

**FIGURE 1:**
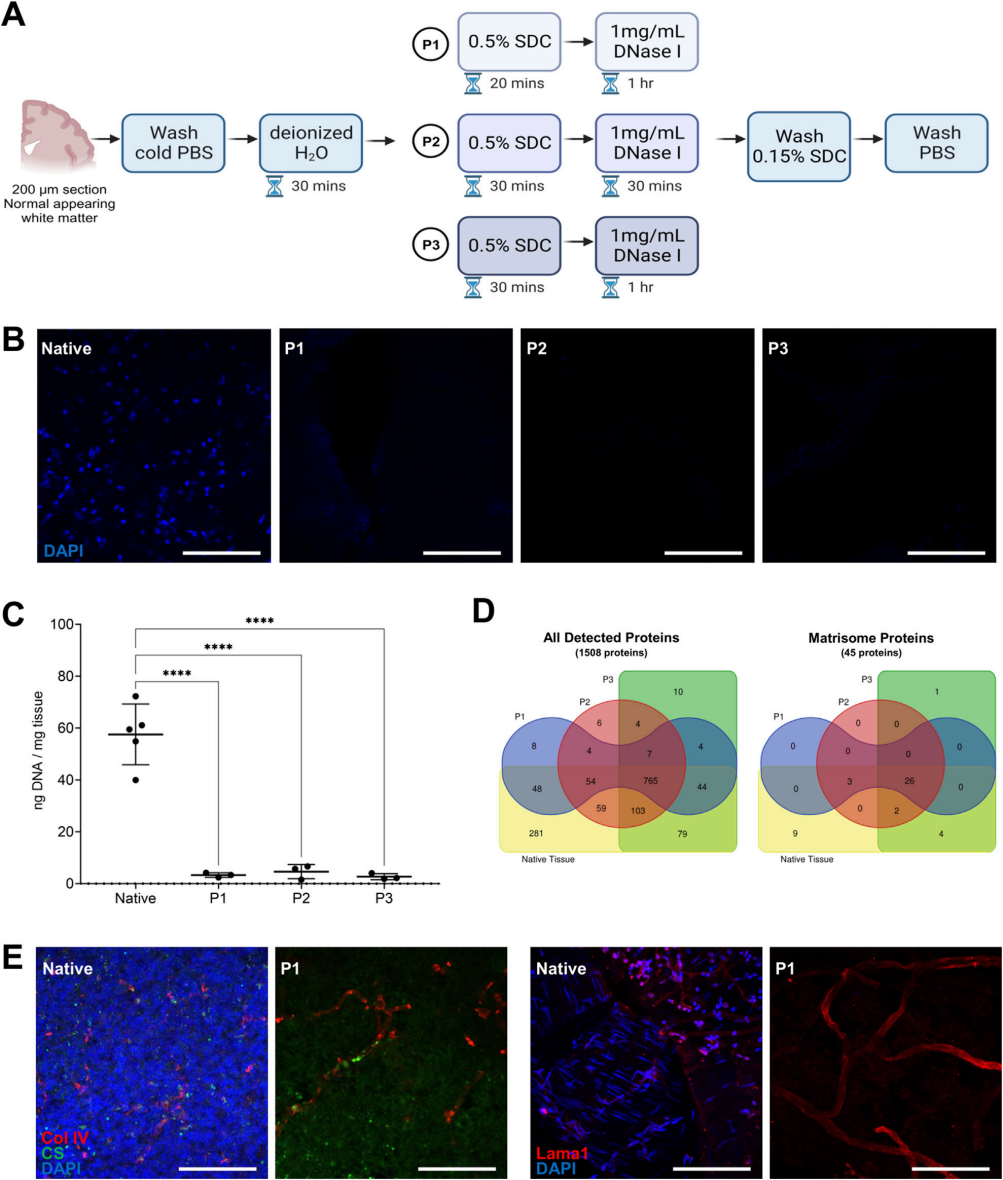
Human brain decellularization protocol. **(A)** Overview of the decellularization method showing the different decellularization protocols (P1, P2, P3) and their different incubation periods. **(B)** Representative images of post-mortem human white matter brain tissue decellularized with P1, P2, P3 protocols as compared to the native tissue. Complete cell removal is observed in all of the protocols. Scale bar: 100 µm. **(C)** DNA quantification for different decellularization protocols (P1, P2, P3) and non-decellularized native tissue. Each dot represents one replicate (n = 5) Turkey’s multiple comparisons test. ****p < 0.0001. **(D)** Venn diagrams showing the number of all detected proteins and matrisome proteins identified in the different decellularization protocols (P1, P2, P3) using post-mortem human white matter brain region. Native human brain white matter was also processed as basis of comparison of non-decellularized sample. There were 45 matrisome proteins analyzed and 26 matrisome proteins shared between the decellularized and non-decellularized samples. **(E)** Representative images of immunohistochemical staining of decellularized and native human brain tissue stained with DAPI, chondroitin sulfate (CS), collagen IV (Col IV), and laminin alpha-1 (Lama1). Scale bar: 100 µm.

We first established a decellularization method for generating DHBTs using 200 µm-thick sections. An overview of the decellularization method is depicted in Figure 1A. The method consists of deionized water to burst the cells, SDC as detergent to wash out cellular components, and DNAse I to remove nuclear contamination. SDC was tested at 0.1% or 0.5% in all investigated regions. Complete decellularization was only achieved at 0.5% SDC (Supplementary Figure S1) and this concentration was then selected to determine the optimal incubation times with detergent and DNase I on normal appearing white matter tissue. We selected 3 decellularization protocols, P1, P2, and P3, to ensure efficient cell removal and maintenance of the protein and glycosaminoglycan composition of the DHBTs. The three protocols are described in detail in the Material and Methods section and overview shown in Figure 1A. In brief, P1 consisted of a 20-min incubation with 0.5% SDC and 1-h incubation with DNase I. P2 consisted of a 30-min incubation with 0.5% SDC and 30-min incubation with DNase I. Finally, P3 combined a 30-min incubation with 0.5% SDC and 1-h incubation with DNase I. To assess cell removal and confirm the efficiency of decellularization, DNA on non-decellularized native tissue and on the decellularized tissue with the 3 protocols using normal appearing white matter was isolated and measured. As shown in Figures 1B,C, all 3 decellularization protocols resulted in a clear reduction of residual DNA compared to the native tissue, as indicated by the lack of DAPI staining. The extracted DNA of the DHBTs presented values between 1.5 ng/mg and 6.7 ng/mg.

We then performed proteomic analysis of the DHBTs decellularized with the 3 different protocols using normal appearing white matter and compared them with non-decellularized native tissue. We used 3 replicates of DHBTs and native tissue from each protocol to assess the protein composition. Proteomic analysis revealed that of the 1508 proteins analyzed, 765 were shared between the decellularized and non-decellularized brain samples. If we focused only on the matrisome proteins, the analysis shows that 26 out of the 45 matrisome-associated proteins analyzed were shared between the decellularized and non-decellularized brain samples. The 3 decellularization protocols led to a similar number of detected proteins with 934 for P1, 1002 for P2, and 1016 for P3. Figure 1D shows the number of proteins detected and shared in the DHBTs from the different decellularization protocols (P1, P2, P3) and native tissue. Next, we tested the three protocols with frontal cortex and SVZ samples. We observed that prolonged incubation periods with SDC (decellularization protocols P2 and P3) were exceedingly stringent for these brain areas, leading to complete degradation of the tissue sections, which could not be used for further investigations. Thus, P1 was selected for the generation of DHBTs from all three brain regions.

To evaluate whether the selected P1 protocol preserves the ECM composition of the DHBTs, three selected core matrisome proteins were investigated by immunohistochemistry (Figure 1E). Collagen IV and laminin alpha-1 were detected in both DHBTs and in non-decellularized control brain tissue. Moreover, we could detect the glycosaminoglycan CS, which builds CS proteoglycans (CSPG) in both the non-decellularized and decellularized tissue after cell removal, which is proven by the absence of DAPI staining.

### 3.2 Proteomic analysis of decellularized tissue from different human brain regions

**FIGURE 2:**
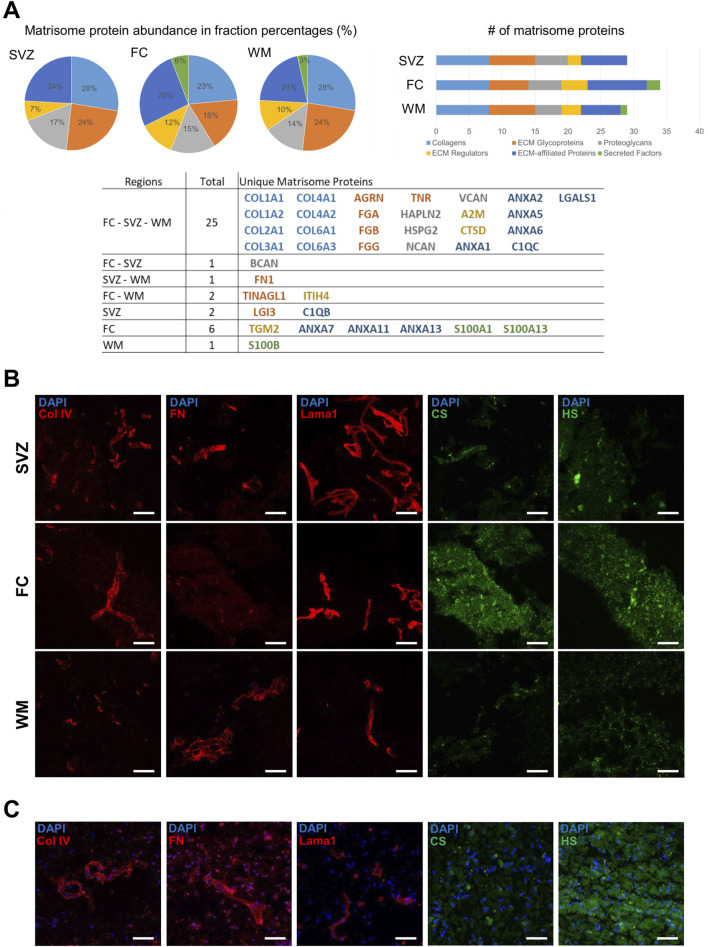
Comparative proteomic analysis of DHBTs. **(A)** Pie charts depict matrisome proteins identified in percentages and bar graphs show absolute numbers of matrisome proteins identified across the region-specific decellularized human brain tissue (DHBT) after proteomic analysis. The table shows the matrisome proteins identified in every region color-coded by the matrisome categories. **(B)** Representative images of immunohistochemical staining for collagen IV (Col IV), fibronectin (FN), laminin alpha-1 (Lama1), chondroitin sulfate (CS), heparan sulfate (HS) as well as DAPI, confirming presence of matrisome proteins and lack of cells across the DHBTs (subventricular zone (SVZ), frontal cortex (FC), and white matter (WM). Scale bar: 50 µm. **(C)** Representative images of immunohistochemical staining of non-decellularized frontal cortex tissue stained with collagen IV (Col IV), fibronectin (FN), laminin alpha-1 (Lama1), chondroitin sulfate (CS), heparan sulfate (HS), and DAPI confirming presence of matrisome proteins in native tissue control. Scale bar: 50 µm.

With the decellularization protocol established for white matter, DHBTs from the frontal cortex and SVZ were generated and characterized by proteomic analysis. The human matrisome proteins from the DHBTs were categorized using the database MatrisomeDB conceived by Naba et al. (2016), Shao et al. (2020). Matrisome proteins were categorized as follows: collagens, glycoproteins, proteoglycans, ECM regulators, ECM-affiliated proteins, and secreted factors. The three brain regions contained similar numbers of detected matrisome proteins (Figure 2A). SVZ and white matter contained 29 ECM proteins, while the frontal cortex contained 34. Based on the categories, all three brain regions shared 8 collagen proteins, 5 glycoproteins, 4 proteoglycans, 2 ECM regulators, and 6 ECM-affiliated proteins. The fibril-forming collagens (types I, II, III), network-forming collagen type IV (COL4A1), and cell-binding collagen type VI (COL6A1, COL6A3) were all present in the three regions-specific DHBTs. Glycoproteins present were agrin (AGRN), fibrinogen chains (FBA, FBB, FBG), and tenascin-R (TNR). Hyaluronan and proteoglycan link protein 2 (HAPLN2) were also observed in all the DHBTs across all the regions. In the proteoglycan family, neurocan (NCAN), versican (VCAN) and heparan sulfate proteoglycan 2 (HSPG2) were present in all the DHBTs.

The table in Figure 2A displays the unique differences observed among the region-specific DHBTs. While brevican (BCAN) was present in the frontal cortex and SVZ, the glycoprotein fibronectin (FN1) was seen only in the SVZ and white matter. The glycoprotein leucine-rich glioma inactivated 3 (LGI3) and an ECM-affiliated protein complement C1q B chain (C1QB) were only present in the SVZ. Unique to the white matter was the secreted factor S100 calcium binding protein B (S100B). Lastly, for the frontal cortex, a number of exclusive proteins included secreted factors S100As, ECM-affiliated annexins, and the ECM regulator TGM2.

To confirm the presence of the main proteins detected by proteomics, immunohistochemistry of key matrisome components such as collagen IV, fibronectin, and laminin alpha-1 was performed. CS or HSPGs were detected by staining of the respective glycosaminoglycans (Figure 2B). All markers are present across all three brain regions except for the lack of fibronectin in the frontal cortex, confirming the proteomic data. The removal of cellular components is evidenced by the lack of DAPI staining. Control native tissue from the FC, which showed high levels of both CS and HS in decellularized modus (Figure 2B), was also stained to validate the presence of the key matrisome components prior to decellularization (Figure 2C). Thus, the composition of the DHBTs retains fundamental matrisome proteins, allowing for the investigation of the effects of the brain ECM on cell seeding, activation and/or differentiation.

### 3.3 Expression analysis of human neural stem cells seeded on region-specific decellularized human brain tissue

**FIGURE 3:**
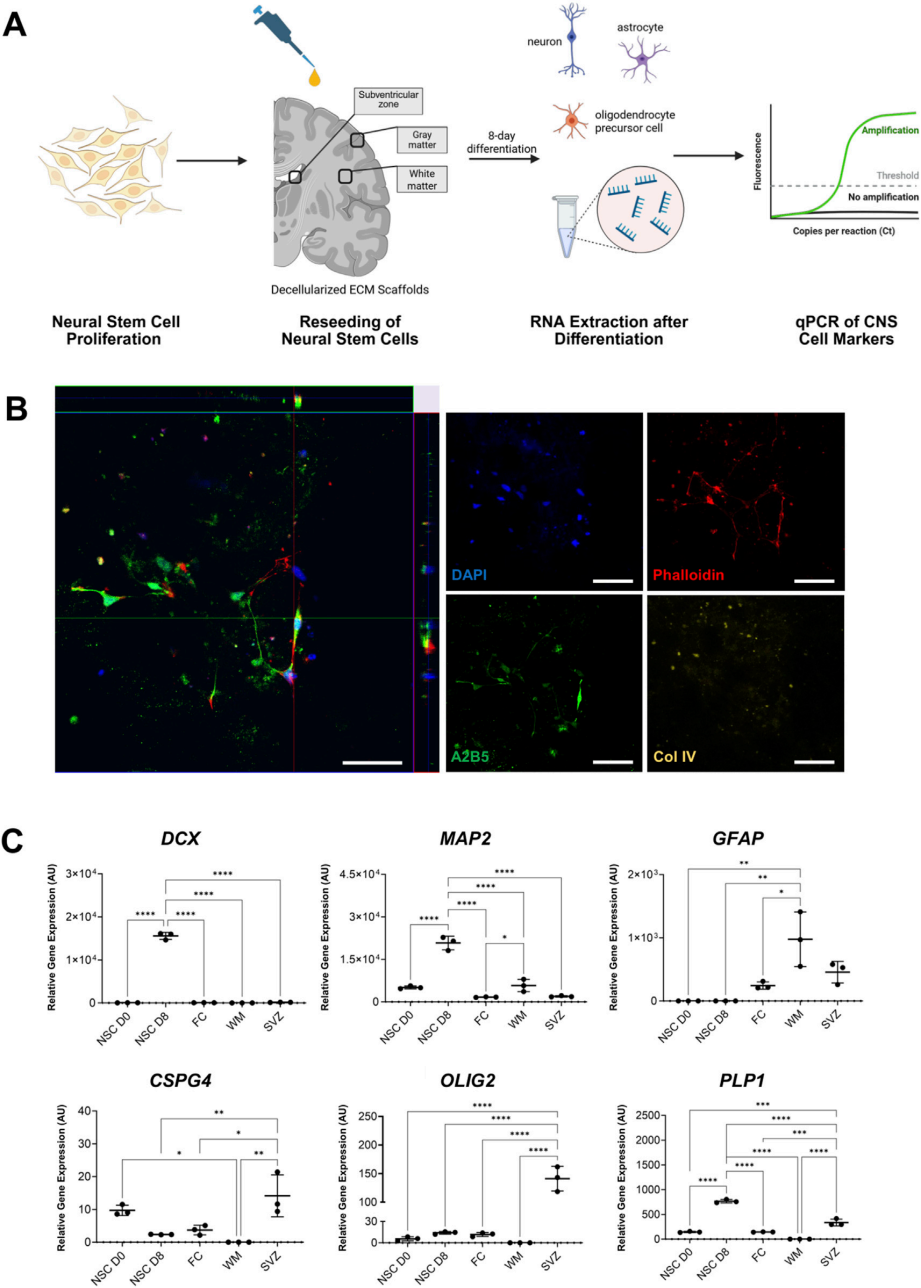
NSCs phenotype after seeding onto regional DHBTs. **(A)** Overview of the neural stem cell seeding on the region-specific decellularized human brain tissue (DHBT). **(B)** Orthogonal view of z-stack images of NSCs (8 days post-seeding) on decellularized human brain tissue with DAPI, phalloidin, A2B5, and collagen IV (Col IV). Scale bar: 50 µm. Image shows NSCs successfully attaching and integrating into the decellularized SVZ tissue and the stem cells cells start expressing A2B5, a marker for OPCs. **(C)** Gene expression analysis of neuronal, astrocytic, and oligodendroglial markers, measured by qPCR in neural stem cells directly before seeding (NSC D0) and after 8-day differentiation in different DHBTs (frontal cortex, white matter, and subventricular zone (SVZ)) and in coated glass (NSC 8-day diff). Each dot represents one replicate (n = 3). Turkey’s multiple comparisons test. *p < 0.05; **p < 0.01; ***p < 0.001, ****p < 0.0001.

To assess the suitability of the region-specific DHBTs to modulate NSCs, we investigated the cell attachment and differentiation of H9 derived NSCs that were seeded onto the DHBTs without further preparation or coating as shown in a schematic diagram in Figure 3A. After NSC seeding, the DHBTs were incubated with serum-free and growth factor-free differentiation medium for 8 days. We observed that the NSCs successfully attached into the DHBT. Moreover, the z-stack images showed that cells survived after seeding as shown by the presence of stem cell nuclei and were well integrated into the DHBT (Figure 3B). ECM structure was visualized by collagen IV staining. Phalloidin staining, which identifies actin filaments, showed cell processes through the DHBTs and between cells. We also stained the recellularized ECM with anti-A2B5 antibodies to identify cell of the glial lineage. As shown in Figure 3B, stem cells, which attached and survived, also differentiated into cells expressing A2B5 after 8 days of differentiation within the DHBTs.

At day 8 after initiation of the culture of NSC with DHBT samples, RNA was extracted and quantified from the viable cells to further assess the gene expression of the expression of different neuronal, astrocytic, and oligodendroglial lineage markers. The stable RNA isolated from the NSCs (NSC D0: 142.5 ng/μL, NSC D8: 105.6 ng/μL, FC: 43.5 ng/μL, SVZ: 68.7 ng/μL, WM: 57.5 ng/μL) indicate cellular stability and viability after 8 days of culture.

Gene expression levels were compared between the three brain regions and a control sample of NSCs that was seeded on glass (NSC D8) as well as with the baseline NSCs prior to seeding and incubation (NSC D0) (Figure 3C). Gene expression analysis showed that the neuronal markers doublecortin (*DCX*) and microtubule-associated protein 2 (*MAP2*) were only detected in the NSC D8 seeded on glass. No change was observed across the region-specific DHBTs for the two neuronal markers. An amplification of glial fibrillary acidic protein (*GFAP*), an astrocytic marker, was observed across all three brain regions. Interestingly, all the oligodendroglial markers, which comprise of chondroitin sulfate proteoglycan 4 (*CSPG4*), myelin proteolipid protein (*PLP1*), and oligodendrocyte transcription factor 2 (*OLIG2*), were all detected exclusively in the SVZ but not in the frontal cortex and white matter. Therefore, the seeded NSCs expressed astrocytic and oligodendroglial markers in the SVZ. On the other hand, cells in the white matter and frontal cortex only upregulated astrocytic markers.

### 3.4 Cytometric analysis of human monocytes seeded on region-specific decellularized human brain tissue

**FIGURE 4:**
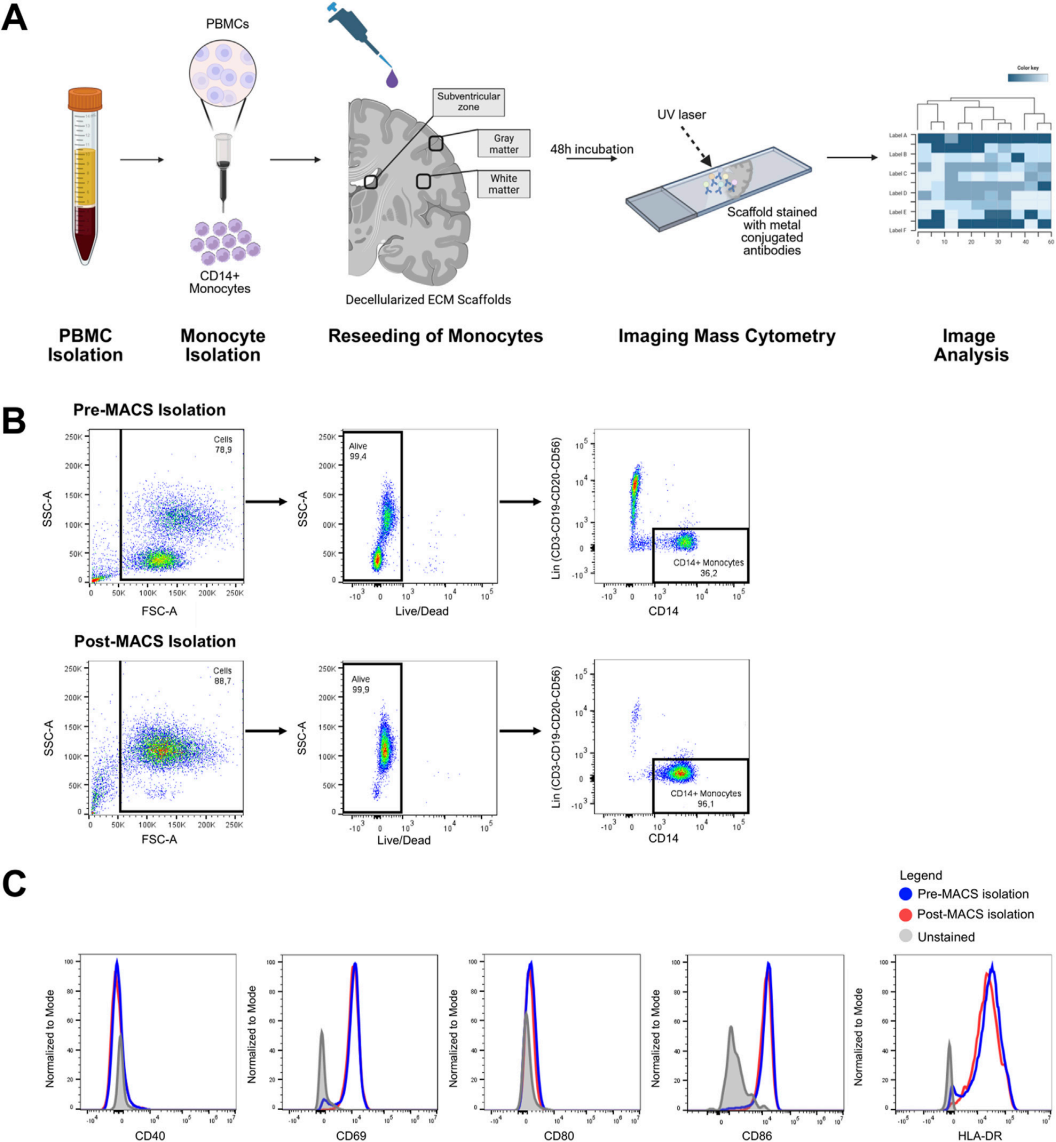
Recellularization of DHBTs with blood-derived monocytes. **(A)** Overview of the peripheral monocyte seeding on the region-specific decellularized human brain tissue (DHBT). **(B)** Flow cytometric gating strategy for CD14^+^ monocytes isolated from PBMCs of a healthy donor. The purity of the monocytes was >90% after magnetic-activated cell sorting. **(C)** Histogram overlay showing the expression of monocyte activation markers before and after the magnetic-activated cell sorting.

To evaluate the suitability of the DHBTs to assess ECM-modulation of peripheral monocytes, MACS-isolated CD14^+^ human monocytes from the peripheral blood of a healthy donor were seeded onto the DHBTs as shown in a schematic diagram in Figure 4A. The purity of the isolated monocytes was determined through flow cytometry, on average above 90% after isolation (Figure 4B). This ensured that only peripheral monocytes were seeded onto the DHBTs and analyzed for the subsequent investigations. To verify if monocytes were activated after isolation and before seeding, the expression levels of the activation markers CD40, CD69, CD80 CD86 and HLA-DR were determined (Figure 4C). Monocytes retained their expression profile pre- and post-MACS isolation, indicating that they were not activated before seeding onto the region-specific DHBTs.

**FIGURE 5:**
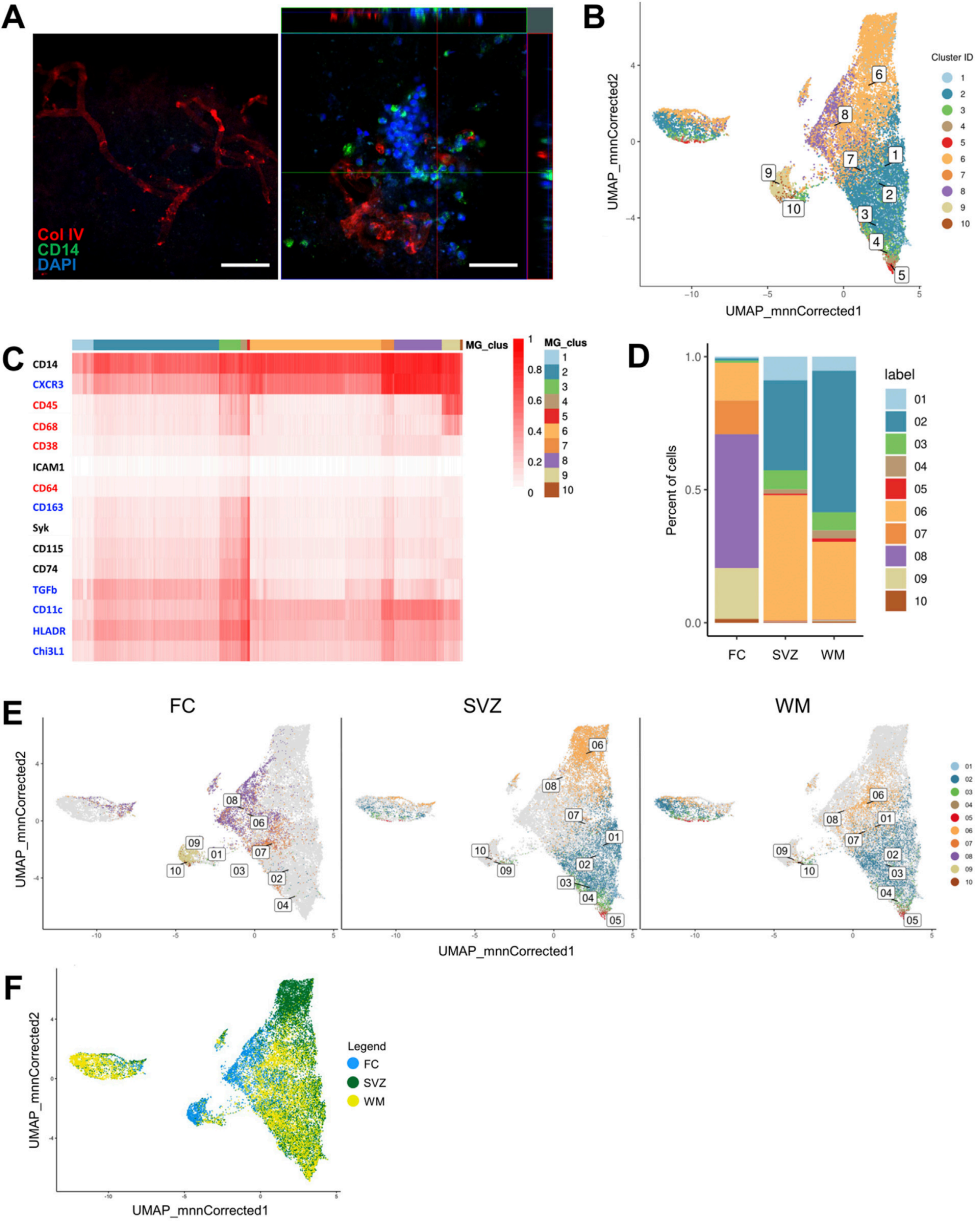
Imaging mass cytometry profiling of monocytes seeded onto DHBTs. **(A)** Orthogonal view of z-stack images showing monocytes 48 h post-seeding on decellularized human brain tissue with CD14, collagen IV (Col IV) and DAPI. Scale bar: 50 µm. **(B)** UMAP based on the arcsinh-transformed expression of 15 markers in the monocyte-derived cells after 48 h in region-specific decellularized human brain tissue (DHBT). The UMAP is color-coded based on the 10 cell population clusters obtained with FlowSOM clustering. **(C)** Heatmap of the median (arcsinh-transformed) expression of 15 markers across the 3 brain regions and 10 cell population clusters. The 15 markers characterize the monocyte-derived cells by anti-inflammatory markers (blue), pro-inflammatory markers (red), and lineage, migration and tissue residency (black). The color in the heatmap represents the median of the arcsinh for each subset (centroids) with 0–1 transformed marker expression. The dendrogram represents the hierarchical similarity for brain regions and clusters (columns) and markers (rows) using hierarchical clustering with Euclidean distance metric and average linkage. **(D)** Subset frequencies for each region-specific DHBT. **(E)** UMAPs based on the frequencies and presence of clusters for each decellularized region-specific DHBT. **(F)** UMAP based on the distribution of the cells color-coded based on brain region: FC (blue), SVZ (green), WM (yellow).

Since the chamber slides were not coated, only the DHBTs acted as a substrate for monocyte attachment. A comparison between decellularized tissue pre and post-recellularization showed that the seeded monocytes successfully attached and survived into the DHBT (Figure 5A). The orthogonal view of z-stack images showed that monocytes integrated into the DHBT after seeding. However, not all of the monocytes retained the CD14 marker, which could indicate differentiation post-seeding (Figure 5A).

In order to unravel the effects of regionally distinct DHBTs on monocytic phenotype, we performed imaging mass cytometry onto the recellularized ECM. For that, the antibody panel was designed to characterize the monocyte-derived cells by their lineage (CD14, CD45), polarization (CD38, CD64, CD68, CD163, CXCR3, CHI3L1, HLA-DR, TGF-beta), and migration and tissue residency (CD11c, CD74, CD115, ICAM1, Syk), and then measured at CyTOF2 (Supplementary Figure S2). To identify the different monocyte populations, we performed clustering analysis with the flowSOM algorithm using the marker expression as input (Van Gassen et al., 2015). Visualization of the multi-dimensional single-cell data using a uniform manifold approximation and projection (UMAP) displayed the distinct 10 cell population clusters with distinct phenotypes in a two-dimensional space (Figure 5B). Heatmap demonstrated the expression levels of all 15 markers used for the cluster analysis amongst the cell population clusters (Figure 5C). Overall, all the defined clusters (1-10) were positive for both CD14 and CXCR3 and had low or no expression of ICAM1. We then analyzed the difference in cluster frequency between regions and found that SVZ and white matter were enriched in clusters 1, 2, 3, 4, 5 (Figure 5D). Clusters 1 and 2 demonstrated an anti-inflammatory phenotype with the expression of CXCR3, TGF-beta, CD11c, HLA-DR, and CHI3L1. The expression of pro-inflammatory markers such as CD68 and CD64, and the migration markers Syk and CD74 characterized clusters 3, 4 and 5. On the other hand, the frontal cortex showed enrichment for clusters 7, 8, 9, 10 (Figure 5D). Cluster 7 had prominent expression of the anti-inflammatory markers CXCR3, TGF-beta, CD11c, HLA-DR, and CHI3L1, and intermediate expression of markers for migration CD74 and Syk. Cluster 8 showed a rather anti-inflammatory profile based on the expression of CXCR3, CD11c, HLA-DR, and CHI3L1. Only clusters 9 and 10 presented a pro-inflammatory phenotype characterized by CD45 and CD68 expression. In addition, cluster 6 with an anti-inflammatory phenotype characterized by the expression of CXCR3, CD11c and HLA-DR, was common to all the three regions (Figure 5D). The similarities between the SVZ and white matter, as well as their differences to the frontal cortex, were demonstrated into three UMAPs showing the distribution and frequency of the 10 cell population clusters in each region (Figure 5E). Additionally, this overlap between the clusters present in the SVZ and white matter was more apparent visually when the monocyte-derived cells in the UMAP were color coded by brain region (Figure 5F). The cells measured in the frontal cortex appeared to not overlap with the other two regions.

## 4 Discussion

In this study, we established a decellularization protocol for human brain tissue that allowed investigations of region-specific matrisomes and the generation of DHBTs suitable to assess effects of local ECM milieu on cellular regenerative processes. Our optimized decellularization protocol is a quick, simple, and straightforward technique that effectively removed cellular and nuclear components (Supplementary Figure S1), maintaining core and associated ECM components. Matrisomes of the SVZ, white matter, and frontal cortex were characterized using proteomics and their effects on the seeding and differentiation of NSCs and blood-derived monocytes were assessed in culture experiments.

As compared to other organs, the human brain tissue has a looser mechanical structure, which presented a challenge in producing ECM scaffolds (Reginensi et al., 2020). We used SDC as it was reported to better preserve collagens and glycosaminoglycans during decellularization (Moffat et al., 2022) as well as cytokines and core proteins in porcine renal scaffolds when compared to other detergents such as SDS and Triton X-100 (Crapo et al., 2011; Fischer et al., 2017). In our hands, the SVZ and cortical sections were more sensitive to SDC than white matter, probably due to their lesser myelin content (Sowell et al., 2003; Corrigan et al., 2021). Therefore, a 20-min SDC incubation time was established as the optimal one for the three investigated regions. After treatment with DNAse I, the DNA content in our DHBTs contained less than 10 ng DNA/mg of tissue, which is below than recommended 50 ng DNA/mg tissue for a successful decellularization (Crapo et al., 2011). In addition, no apparent nuclei were observed with DAPI staining, while matrix components, such as collagen IV, CS and laminin alpha-1, remained in the DHBTs. Compared with other decellularization protocols applied to brain tissue, our protocol was simple, fast and effective for human brain sections. For instance, De Waele et al. (2015) developed a 3-cycle protocol with SDC and Triton-X for mouse brain sections, while the protocol developed by DeQuach et al. (2011) required 3–4 days incubation with SDS.

In the context of tissue engineering applications, defining the assembly of decellularized organotypic human brain ECM scaffolds is fundamental. The proteomic analysis of our DHBTs confirmed the presence of ECM proteins, which are indispensable for cell adhesion and in influencing cell behavior, especially for recellularization assays. These included collagens, glycoproteins, proteoglycans, ECM regulators, ECM-affiliated proteins, and secreted factors. Fibril-forming collagens (types I, II, III), network-forming collagen IV, and cell-binding collagen type VI were present. In addition, glycoproteins detected included AGRN, fibrinogen chains, and TNR. AGRN together with laminin, is an essential structural protein of the basal lamina in the brain (Hoddevik et al., 2020). The fibrinogen subunits (FGA, FGB, and FGG) polymerize to form the fibrin, which is involved in cell adhesion (Golanov et al., 2019), while TNR, which is exclusively expressed in the CNS, functions as a proteoglycan crosslinker involved in cell adhesion and outgrowth (Wagner et al., 2020). Also, the CSPGs NCAN and VCAN as well as HSPG2 are important for cell adhesion and intracellular signaling (Bonneh-Barkay and Wiley, 2009) or stabilization of the basement membrane (Reed et al., 2019), respectively. HAPLN2 stabilizes the interactions between hyaluronan and essential proteoglycans in the ECM for efficient neuronal conductivity (Wang et al., 2019). In our study, high presence of HS and CS were confirmed in the decellularized FC and also in relatively lower amount in FC native tissue. On the other hand, the SVZ and WM native tissues showed almost undetectable amounts of GAGs, in line with the low detection observed in these decellularized regions. Compared with the dataset from Cho et al. (2021), Cho et al. (2021), we showed that the different collagen types were more abundant than glycoproteins, representing 23%–28% of the ECM proteins. We hypothesize that the low presence of glycoproteins and proteoglycans could derive from factors such as sample size, decellularizing agent used, and/or the method used for protein detection.

Even though the three brain regions showed no significant differences in the total numbers of detected matrisome proteins, proteomic analysis identified unique differences. BCAN, a CSPG, which are involved in neurite growth and synaptic function (Bonneh-Barkay and Wiley, 2009), were present in the frontal cortex and SVZ, while FN1 was detected only in the SVZ and white matter. FN1 is also a vital component for cell adhesion, growth, and differentiation (Griffiths et al., 2020). The glycoprotein LGI3 and an ECM-affiliated protein C1QB were only seen in the SVZ. In the brain, LGI3 is exclusively expressed by oligodendrocytes and seems to be involved in neuronal exocytosis and regulation of synaptic plasticity (Lee et al., 2006; Cahoy et al., 2008; Miyazaki et al., 2024). On the other hand, the complement protein C1QB seems to be relevant for synaptic pruning during development and in processes of aging and degeneration (Cho, 2019), but not much is known about its role in the SVZ. On the other side, the Ca^2+^ binding protein S100B, which is highly expressed by astrocytes and related to inflammation, was only present in the white matter region (Michetti et al., 2023). The frontal cortex was characterized by a number of unique proteins such as the S100As, annexins, and transglutaminase 2 (TGM2). The annexin family and S100A associated proteins are regulators of different calcium-dependent cellular processes and secreted members of the S100A family appear to influence neuronal activity (Donato et al., 2013; Zhang et al., 2021). There were three annexins uniquely present in the frontal cortex, namely, ANXA7, ANXA11, and ANXA13, which are involved in cellular growth and various signal transduction pathways (Gerke et al., 2024). TGM2 is associated with cell growth and differentiation, ECM assembly, and tissue repair (Kanchan et al., 2015). It is worth noting that factors defined as secreted may have a primary cellular origin and are now present or have an increased presence in the DHBTs because of their release from the intracellular space during cell membrane lysis.

Overall, although it remains speculative, the comparative proteomic data of these three brain regions point to a rather plasticity-promoting profile of the SVZ DHBTs and a neuronal activity-modulating profile of the cortical and, partly, of the WM DHBTs. Decisively, proteomic data from human decellularized ECM will be instrumental in constituting a matrisome map of the human brain. Even with the new expansion of MatrisomeDB 2.0 offered by Shao et al., only information on the rodent brain is available and there is still a lack of matrisome data on the human brain (Shao et al., 2023).

Decellularized ECM scaffolds or ECM-based biomaterials have been employed for different culture-based and tissue engineering applications for investigating regenerative approaches (Yaldiz et al., 2022; Golebiowska et al., 2024). However, generally, coating substrates containing collagen, laminin, or poly-d-lysine or protein mixtures such as matrigel and geltrex composed of collagen IV, HSPGs, laminin, and growth factors are utilized as cell culture substrates to facilitate cell attachment or to promote neuronal growth (Kim et al., 2011; Liu et al., 2020). These alternatives inherently present disadvantages such as complexity in production, batch variability, and inability to fully replicate the highly complex native ECM (Aisenbrey and Murphy, 2020; Nicolas et al., 2020). This study demonstrated that some of these challenges were overcome using our model, which also permits to evaluate how distinct ECMs may affect cells differentially.

To characterize the phenotype of the original NSC after 8 days incubation within the DHBTs, lineage markers for each cell type were selected. Analysis of seeded NSCs, indicated the presence of cells expressing the astrocytic marker *GFAP* within the white matter and frontal cortex DHBTs. Alternatively, in the SVZ, the cells upregulated not just the astrocytic marker, but also the oligodendroglial markers *CSPG4*, *PLP1*, and *OLIG2*. The expression of neuronal markers was only observed *in vitro* when the NSCs were directly seeded onto the coated glass chambers. Our data seems to indicate that ECM from distinct brain regions act differentially on NSC differentiation. Spontaneous differentiation of NSCs into neuronal cells after seeding on glass surface had been previously reported (Ostrakhovitch et al., 2012; Taylor et al., 2019). Within the DHBTs, however, the NSCs rather favored oligodendroglial and astrocytic differentiation. We speculate therefore that the unique ECM composition of the DHBTs promoted gliogenesis. Tissue or substrate stiffness has been shown to influence neurite growth and neurogenesis, and it is known that stiffness is increased in neurogenic niches in the brain (Kjell et al., 2020). It is also well established that the SVZ promotes NSC differentiation towards different CNS cell types including oligodendrocyte precursors and mature-myelinating oligodendrocytes (Zywitza et al., 2018). Importantly, our data indicates that this intrinsic ability of the SVZ to generate cells of the oligodendroglial lineage is retained by SVZ decellularized ECM, perhaps mediated by LGI3, which was only detected within the SVZ and was reported to direct the NSCs to an oligodendrocyte lineage (Miyazaki et al., 2024).

We next demonstrated the suitability of the DHBTs to assess effects mediated by the ECM on human monocytes. Monocytes successfully attached, integrated, and differentiated into the DHBTs. The monocyte population clusters present in the SVZ and white matter overlapped and included the most frequent anti-inflammatory clusters 1 and 2, and the intermediate clusters 3 and 6, which showed both anti- and pro-inflammatory characteristics. The similar phenotype of the seeded monocytes could be explained by the similar ECM composition and high myelin content of the SVZ and white matter (Zywitza et al., 2018). In line with this, myelin phagocytosis by macrophages has been proven to induce an anti-inflammatory phenotype of myelin-laden monocyte-derived dendritic cell (Gredler et al., 2010). As indicated by the proteomic data, the presence of fibronectin in the SVZ and white matter might have also influenced the anti-inflammatory polarization of monocytes. Fibronectin rich-tissues have been shown to promote the phagocytosis of debris by macrophages (Griffiths et al., 2020) and to influence regenerative cellular responses in injured tissues through their integrin-binding domain (Bachman et al., 2015). In the frontal cortex, however, anti-inflammatory clusters 7 and 8, and the pro-inflammatory cluster 9 are more frequent. Proteomic data showed the expression of TGM2 only in this region, which was shown to induce both the pro- and anti-inflammatory polarization of macrophages (Sun and Kaartinen, 2018). Taken together, most of the population clusters of the seeded monocytes showed an anti-inflammatory profile, which is in line with previous studies that reported that ECM hydrogels derived from porcine optic nerve, spinal cord, and brain tissue promoted pro-remodeling and anti-inflammatory macrophage phenotype (Dziki et al., 2017; Hong et al., 2020). Although this data needs to be validated in future experiments, our results represent an exemplary application of the DHBTs for regenerative medicine.

Overall, we successfully established and optimized a decellularization protocol in generating decellularized ECM from human brain tissue from three different brain regions. The decellularization process gave way to the proteomic characterization of region-specific DHBTs, expanding the knowledge of matrisome proteins present in the human brain. The DHBTs showed support in cell adhesion, survival, and phenotype differentiation of seeded monocytes and NSCs. Future studies can evaluate the interactions between the region-specific DHBTs and different cell types in terms of cell viability, proliferation, migration, and downstream differentiation.

As a proof-of-concept investigation, the present study has limitations. Tissue samples of the three brain regions were derived from a single donor. Additionally, comparison with healthy control brain samples needs to be included in future investigations. The limited amount of autopsy material supplied also imposed a challenge in generating sufficient brain sections for further characterization of mechanical properties of the DHBTs. Furthermore, the biological characteristics of the donor such as age, sex, or health conditions may influence the composition and functionality of the DHBT generated in this study. With aging, the brain ECM undergoes significant biochemical changes including collagen reduction, GAG remodeling, and increase of cross-linking, which alter the structural and mechanical properties of the matrix (Soleman et al., 2013; Foscarin et al., 2017; Hebisch et al., 2023). Sex-related differences in brain ECM composition, specifically collagen type IV, fibronectin, and laminin have been investigated using healthy and multiple sclerosis mouse models (Batzdorf et al., 2022). The donor in the study has PSP, a neurodegenerative tau-related pathology that may alter ECM composition through increased expression of ECM-modifying enzymes (Bonneh-Barkay and Wiley, 2009) and pro-inflammatory cytokines, and/or altered glycosylation patterns (Moretto et al., 2022). Though these alterations related to age, gender, or disease state may influence the observed proteomic profiles of the region-specific DHBTs and the behavior of the seeded cells, the decellularization protocol and the comparison between distinct brain regions in terms of protein profile and ability to modulate both myeloid cells and neural stem cells are highly reliable. Our findings will significantly contribute to design future studies using decellularized tissue to address the impact of region-specific ECM on tissue regeneration and to understand how brain pathologies may modulate or change these effects.

In conclusion, granting the challenges in developing ECM scaffolds, particularly from the human brain, for analytical and engineering approaches, the decellularization protocol successfully developed in this study generated region-specific DHBTs that maintained both core matrisome and matrisome-associated proteins. This ECM model is suitable for investigating cell-matrix interactions. Specifically, DHBTs were applied for recellularization and cell-based assays, examining how neural stem cells differentiated into different CNS cell types and how different monocyte phenotypes are induced depending on the brain region of origin. This project contributes to the development and utilization of ECM scaffolds derived from the human brain. Although further studies are still required to enhance the understanding of the human brain extracellular matrix and its regenerative properties. Taken together, our model could enable the identification of ECM-related therapeutic targets and novel signaling pathways, facilitating efficient regeneration in the CNS.

## Data Availability

The datasets presented in this study can be found in online repositories. The names of the repository/repositories and accession number(s) can be found below: http://www.proteomexchange.org/, PXD062743.
