# Correction: Generation of decellularized human brain tissue for investigating cell-matrix interactions: a proof-of-concept study

**DOI:** 10.3389/fbioe.2025.1690982

**Published:** 2025-09-09

**Authors:** Roemel Jeusep Bueno, Camila Fernández-Zapata, Maren Salla, Juliana Campo Garcia, Aylin Alacam, Oliver Klein, Chotima Böttcher, Helena Radbruch, Friedemann Paul, Sarah C. Starossom, Rafaela V. Silva, Carmen Infante-Duarte

**Affiliations:** ^1^ Experimental and Clinical Research Center, A Cooperation Between Max Delbrück Center and Charité Universitätsmedizin Berlin, Berlin, Germany; ^2^ Faculty of Life Sciences, Humboldt-Universität zu Berlin, Berlin, Germany; ^3^ Berlin School for Regenerative Therapies (BSRT), Charité – Universitätsmedizin Berlin, Berlin, Germany; ^4^ Max-Delbrück-Center for Molecular Medicine (MDC), Berlin Institute for Medical Systems Biology (BIMSB), Berlin, Germany; ^5^ Berlin Institute of Health at Charité Universitätsmedizin Berlin, Berlin, Germany; ^6^ Department of Biology, Chemistry, Pharmacy, Freie Universität Berlin, Berlin, Germany; ^7^ Berlin Institute of Health (BIH), Center for Regenerative Therapies, Berlin, Germany; ^8^ Department of Neuropathology, Charité-Universitätsmedizin, Berlin, Germany

**Keywords:** brain extracellular matrix, matrisome, neuronal stem cells, monocytes, brain proteomics, imaging mass cytometry

In the **acknowledgements**, a sentence acknowledging the technical support of the BIH Cytometry Core Facility and the BIH Imaging Mass Spectrometry Unit and the support of Berlin-Brandenburg School for Regenerative Therapies PhD Program should be included.

The correct **Acknowledgements** is:

“The authors acknowledge the technical support provided by the BIH Cytometry Core Facility and the BIH Imaging Mass Spectrometry Unit and the support of the Berlin-Brandenburg School for Regenerative Therapies PhD Program during the whole period of the study. The authors are most grateful to the individuals and their relatives for consenting to autopsy and subsequent research, which were facilitated by the Charité Neuropathology Biobank.”

The original article has been updated.

