# Supplementary figures and images for: Generation of decellularized human brain tissue for investigating cell-matrix interactions: a proof-of-concept study
Source: Front Bioeng Biotechnol. 2025 Jun 5;13:1578467. doi: 10.3389/fbioe.2025.1578467 (PMC12177465; doi:10.3389/fbioe.2025.1578467)

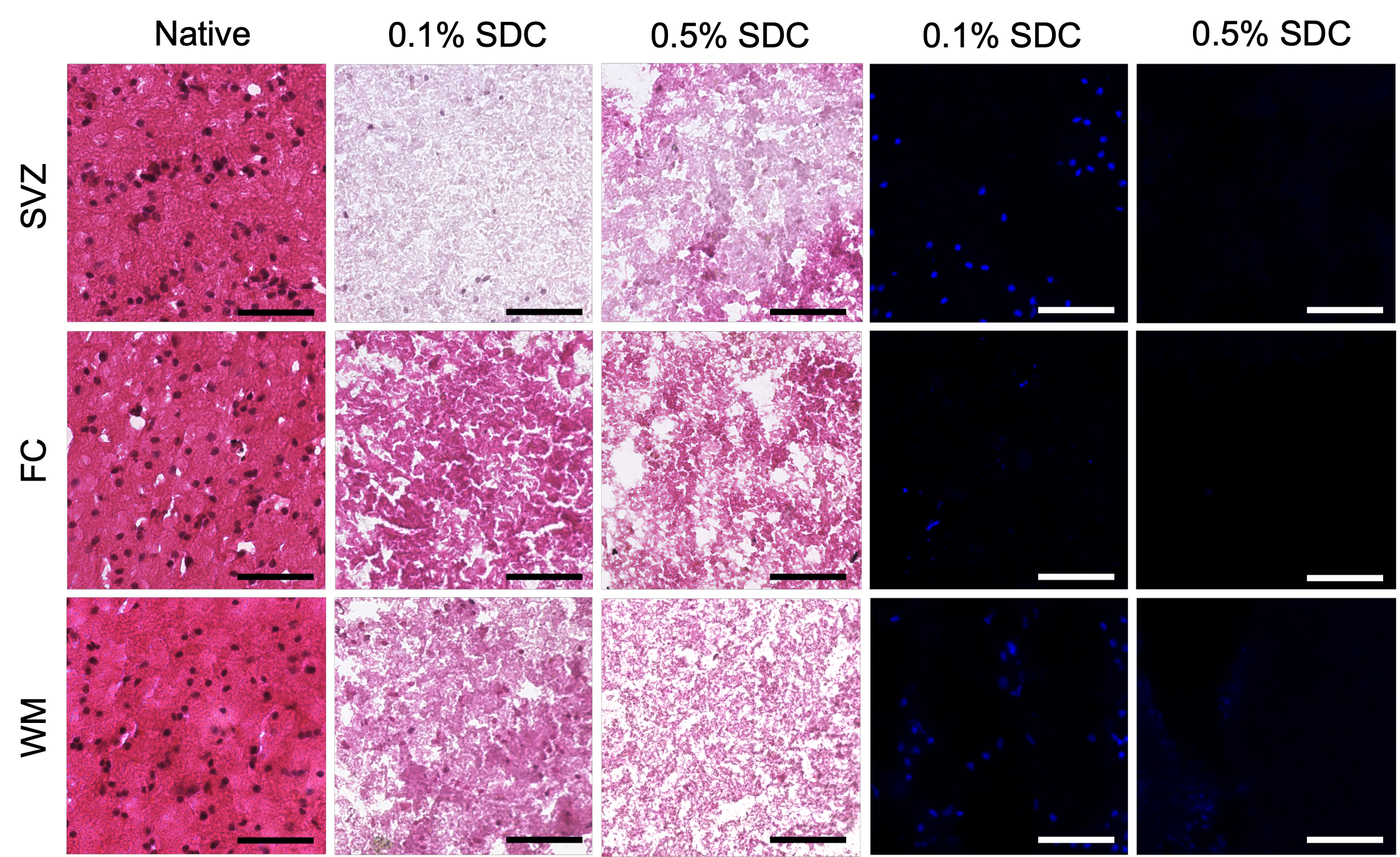

Supplement: Supplementary file 1 [file Image1.tiff]

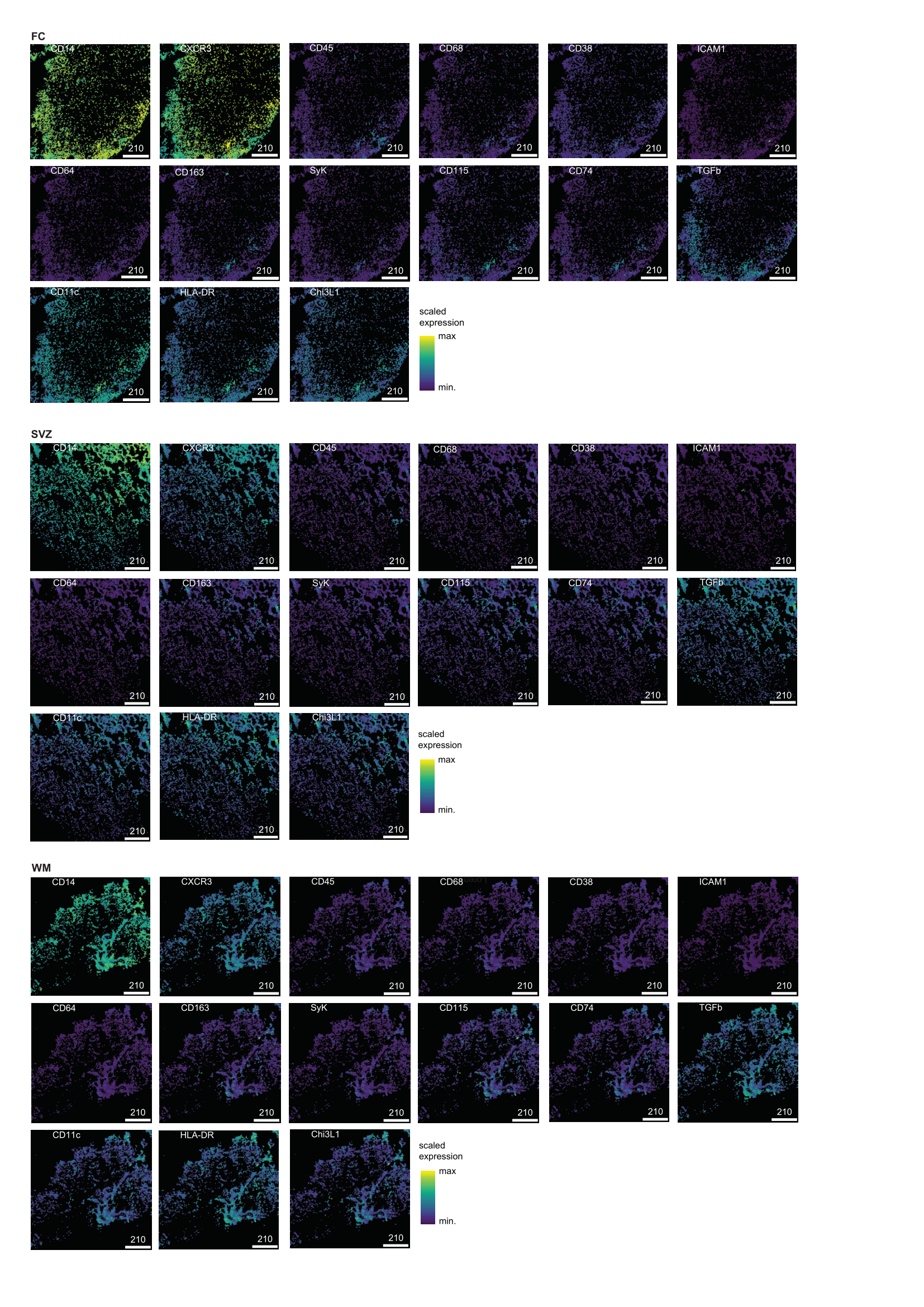

Supplement: Supplementary file 2 [file Image2.tiff]
