# Supplementary material for: Generation of decellularized human brain tissue for investigating cell-matrix interactions: a proof-of-concept study
Source: Front Bioeng Biotechnol. 2025 Jun 5;13:1578467. doi: 10.3389/fbioe.2025.1578467 (PMC12177465; doi:10.3389/fbioe.2025.1578467)
Supplement: Supplementary file 3 [file DataSheet1.docx]

**Supplementary Material**

| Supplementary Table S1. qPCR primer set | | | |
| --- | --- | --- | --- |
| Primers | Assay ID | Amplicon size (bp) | Company |
| *B2M* | Hs00187842_m1 | 64 | Thermo Fisher Scientific, USA |
| *CSPG4* | Hs00426981_m1 | 113 | Thermo Fisher Scientific, USA |
| *DCX* | Hs00167057_m1 | 77 | Thermo Fisher Scientific, USA |
| *GFAP* | Hs00909233_m1 | 57 | Thermo Fisher Scientific, USA |
| *MAP2* | Hs00258900_m1 | 98 | Thermo Fisher Scientific, USA |
| *OLIG2* | Hs00377820_m1 | 64 | Thermo Fisher Scientific, USA |
| *PLP1* | Hs00166914_m1 | 61 | Thermo Fisher Scientific, USA |

| Supplementary Table S2. IMC Antibody Panel | | | |
| --- | --- | --- | --- |
| Target | Isotope tag | Clone | Company |
| CD38 | ^141^Pr | EPR4106 | Abcam, UK |
| ICAM1 | ^142^Nd | HA58 | BioLegend, USA |
| CD14 | ^144^Nd | EPR3653 | StandardBio, USA |
| CD64 | ^146^Nd | 10.1 | StandardBio, USA |
| CD163 | ^147^Sm | EDHu-1 | StandardBio, USA |
| Syk | ^149^Sm | 4D10.2 | StandardBio, USA |
| CD45 | ^152^Sm | D9M8I | StandardBio, USA |
| CD11c | ^154^Sm | Polyclonal | StandardBio, USA |
| CXCR3 | ^156^Gd | G025H7 | StandardBio, USA |
| CD68 | ^159^Tb | KP1 | StandardBio, USA |
| CD115 | ^164^Dy | 9-4D2-1E4 | BioLegend, USA |
| CD74 | ^166^Er | LN2 | StandardBio, USA |
| HLA-DR | ^174^Yb | YE2/36 HLK | StandardBio, USA |
| Chi3L1 | ^176^Yb | EPR19078-157 | Abcam, UK |
| DNA | ^191^Ir | - | StandardBio, USA |
| DNA | ^192^Ir | - | StandardBio, USA |

**Supplementary Figure Captions**

**Supplementary Figure 1**. Representative images from hematoxylin and eosin–stained and DAPI-stained sections of native and decellularized human brain tissue using 0.1% and 0.5% sodium deoxycholate (SDC) from the three different brain regions: subventricular zone (SVZ) frontal cortex (FC), and white matter (WM). Scale bar: 50μm. Note the presence of cells in the tissue decellularized with 0.1% SDC indicating unsuccessful decellularization and the absence of cells from the tissue structure of decellularized human brain tissue using 0.5% SDC indicating successful tissue decellularization of the three brain regions.

**Supplementary Figure 2.** Representative images from imaging mass cytometry staining for the 15 target markers across all three brain regions. Scale bar: 210µm.
